# Mining for single nucleotide polymorphisms in pig genome sequence data

**DOI:** 10.1186/1471-2164-10-4

**Published:** 2009-01-06

**Authors:** Hindrik HD Kerstens, Sonja Kollers, Arun Kommadath, Marisol del Rosario, Bert Dibbits, Sylvia M Kinders, Richard P Crooijmans, Martien AM Groenen

**Affiliations:** 1 Animal Breeding and Genetics Group, Wageningen University, PO Box 9101, Wageningen, 6701 BH, the Netherlands; 2 IPG, Institute for Pig Genetics, PO Box 43, Beuningen, 6640 AA, the Netherlands

## Abstract

**Background:**

Single nucleotide polymorphisms (SNPs) are ideal genetic markers due to their high abundance and the highly automated way in which SNPs are detected and SNP assays are performed. The number of SNPs identified in the pig thus far is still limited.

**Results:**

A total of 4.8 million whole genome shotgun sequences obtained from the NCBI trace-repository with center name "SDJVP", and project name "Sino-Danish Pig Genome Project" were analysed for the presence of SNPs. Available BAC and BAC-end sequences and their naming and mapping information, all obtained from SangerInstitute FTP site, served as a rough assembly of a reference genome. In 1.2 Gb of pig genome sequence, we identified 98,151 SNPs in which one of the sequences in the alignment represented the polymorphism and 6,374 SNPs in which two sequences represent an identical polymorphism. To benchmark the SNP identification method, 163 SNPs, in which the polymorphism was represented twice in the sequence alignment, were selected and tested on a panel of three purebred boar lines and wild boar. Of these 163 in silico identified SNPs, 134 were shown to be polymorphic in our animal panel.

**Conclusion:**

This SNP identification method, which mines for SNPs in publicly available porcine shotgun sequences repositories, provides thousands of high quality SNPs. Benchmarking in an animal panel showed that more than 80% of the predicted SNPs represented true genetic variation.

## Background

Single nucleotide polymorphisms (SNPs), one of the most abundant types of sequence polymorphisms in the genome, are the most suitable markers for genetic linkage mapping, fine-mapping and haplotype reconstruction. Over the past decade, SNPs have been the marker of choice due to their high stability, density and the highly automated way in which SNPs are detected and SNP assays are performed. However only a limited number of SNPs have been identified in the pig, a species of considerable economical and medical importance. A few thousand SNPs in the pig are currently available, and these were mainly identified in expressed genes by either in vitro techniques [1] or by mining porcine expressed sequence tag (EST) sequence databases [2,3]. In humans, the large-scale identification and characterization of SNPs has attracted much more attention, and consequently over 14 million SNPs (dbSNP build 128) have been identified [4], 3.1 million of which have been genotyped SNPs [5] and the SNP density is estimated as one SNP per 1000–2000 bases [6]. Genome scans with high SNP densities have proven to be an effective tool in whole genome association studies to identify genes involved in complex genetic traits [7-10]. The SNP density in pigs is about four-fold higher than that in humans with SNPs found at, on average, every 300 to 400 bps [11]. Despite the availability of the most highly continuous bacterial artificial chromosome (BAC) map of any mammalian genome [12] and the ongoing sequencing efforts in the pig [13], no large scale SNP mining on pig genome sequences has been published. The lack of a pig genome draft assembly still hampers the traditional method of identifying SNPs, in which DNA shotgun sequences of different individuals are aligned to a genomic region of interest using alignment algorithms [14]. In these alignments, sequences are easily compared and SNP candidates can be reliably detected by computational methods like PolyPhred [15], which has been extensively tested for human SNP discovery [16-18]. Despite the unavailability of a draft sequence of the pig genome, a wealth of high quality sequence and mapping data is publicly available that can be used for SNP detection purposes.

Here we describe a high throughput genome sequence mining pipeline from data of the ongoing pig genome sequencing project. With this approach, we performed a SNP mining analysis on the whole genome shotgun dataset generated by the Danish-Chinese Pig Genome Sequencing Initiative [19] that is publicly available in the NCBI Trace Archive. BAC sequence data and the BAC mapping information to the porcine physical map [12] were combined and we used this as a crude assembly of a reference genome sequence. The pipeline is built from existing public software packages and implemented on a computer cluster, which enables efficient mining of large sequence data sets in parallel.

The encouraging outcome of this study is a good starting point for the development of a rapidly growing genome-wide set of SNP markers in the pig.

## Results

### Clustering

At completion of this analysis, the number of finished and contigs of unfinished porcine BAC sequences was 318 and 84,017, resulting in 50,225,986 and 1,164,409,065 total nucleotides, respectively. The NCBI Trace repository contained 4,774,371 whole genome shotgun sequences for center SDJVP, with a total of 3,478,199,073 nucleotides.

**Figure 1:**
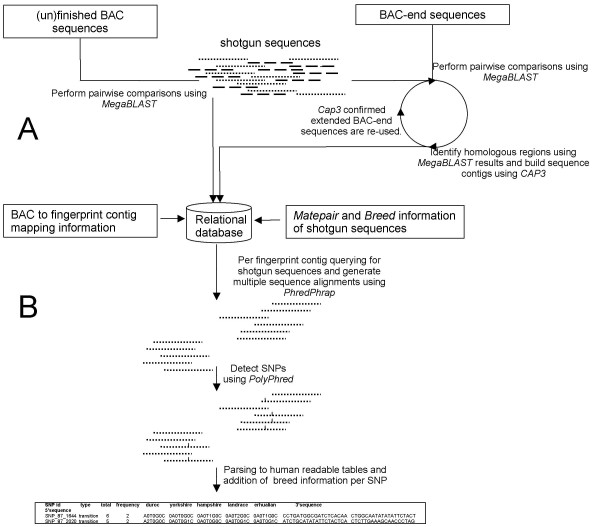
**Steps performed for SNP mining in whole genome shotgun sequences in which BAC(-end) sequences and their mapping information served as a reference genome**. Initially shotgun sequences are assigned to fingerprint contigs (A). Subsequently per fingerprint contig SNPs were mined using PhredPhrap and PolyPhred (B).

Because the analysis of the complete data set for the whole genome was computationally too demanding, the identification of SNPs was performed by a 2-step process. First, the shotgun sequences were assigned to a fingerprint contig by clustering based on their sequence similarity to BAC and BAC-end sequences. The results of the clustering by alignment were stored in a relational database. The BAC and the BAC-end naming as well as the mapping data provided the necessary information to assign the obtained sequence clusters to a specific fingerprint contig on the porcine physical BAC map. Clustering of the shotgun reads with BAC or BAC-end sequences is outlined in Figure 1A. This approach enabled the chromosomal assignment of the sequences, even for chromosomes and chromosomal regions for which currently no assembled chromosome sequence is available at the pre-ensemble [20] website. In total, 838,711 shotgun sequences were clustered and assigned to a specific fingerprint contig (fpc) and 97.7% of these shotgun sequences mapped to a single unique fpc.

### Identifying candidate SNPs

In the second step, the actual identification of SNPs was performed per fingerprint contig. In this respect, a fingerprint contig can be considered a 'genomic region of interest', which is the starting point in traditional SNP mining in species for which a genome draft is available. Per fingerprint contig, the relational database was queried for shotgun reads, in which repetitive sequences were tagged, and were aligned using PhredPhrap [21-23]. Finally, the alignments were searched for SNPs using PolyPhred [15] as outlined in Figure 1B. Identified SNPs were categorised by the number (one, two, three or four) of sequences that represent identical nucleotide substitution on the SNP position in the sequence alignment. SNP prediction results of all fingerprint contigs were combined and analysed for redundancy. Redundancy was expected, because a small fraction (2.3%) of the shotgun reads did not uniquely map to a single fingerprint contig. Paralogous and repetitive sequences typically cause ambiguous clustering results. Although the initial clustering of shotgun sequences was refined in the alignment procedure by Phrap [23], a small number of SNPs still mapped to two distinct genomic regions. These ambiguous SNPs were removed from the data, resulting in a final list of 98,151 unique SNPs (Table 1). The number of identified SNPs was drastically reduced when the constraint for the number of sequences representing identical nucleotide substitution in a SNP was increased. When this number was raised above two, the majority of predicted SNPs were located within a genomic context that was tagged as repetitive sequence.

**Table 1:** SNPs identified, substitution ratios and the fraction in repetitive context at increasing polymorphism representation constraints.

**SNP representation**	**Total SNPs identified**	**Transition/transversion**	**Fraction SNPs in repetitive sequence**
1	98151	1.9	0.39
2	6374	2.8	0.60
3	1202	4.2	0.90
4	462	5.8	0.96

### Distribution of SNPs over the pig genome

At completion of this analysis (Dec 2007), the sequencing of the pig genome was ongoing and most assembled BAC contig sequences were available for chromosomes 1, 4, 7, and 14 The number of SNPs as a percentage of total number of identified SNPs per analysed chromosome is provided in Table 2.

**Table 2:** Distribution of SNPs over the analysed pig chromosomes in percentages of the total number identified.

**SNP**	**Chromosome**																
**representation**	**1**	**2**	**3**	**4**	**5**	**7**	**8**	**9**	**11**	**12**	**13**	**14**	**15**	**16**	**17**	**18**	
1	20,2	3,0	2,2	12,7	2,9	12,7	2,4	1,0	5,9	0,3	6,2	15,5	6,2	2,2	5,1	1,4	
2	22,6	2,6	2,0	12,1	2,2	14,1	2,0	1,0	4,4	0,2	5,7	16,5	7,4	1,8	4,0	1,3	

**Figure 2:**
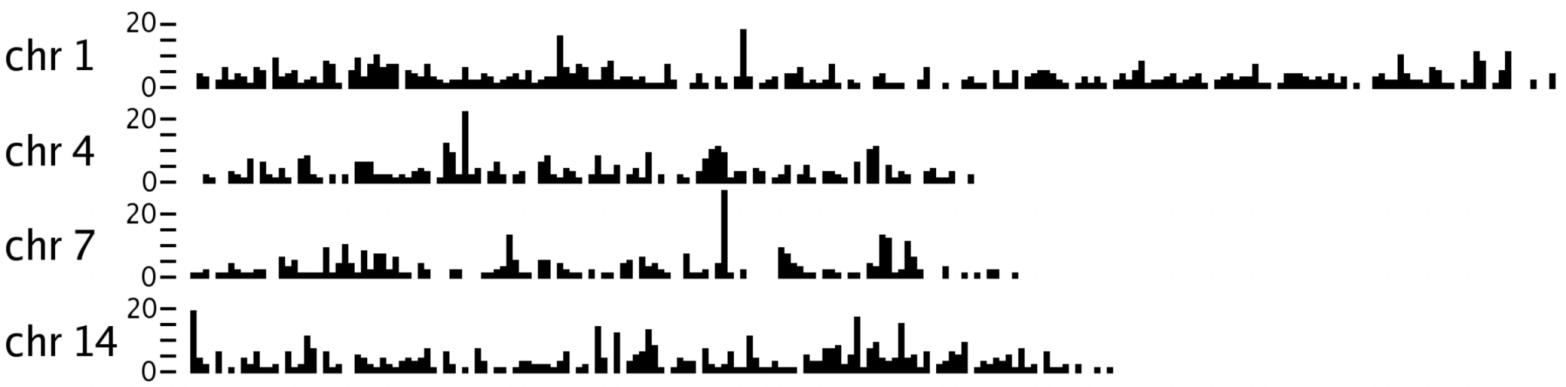
**Distribution of SNPs on pig chromosomes 1, 4, 7 and 14**. The X-axis represents the chromosome in intervals of 1 Mb in size. On the Y-axis the number of identified SNPs is shown for the 1 Mb intervals, each tick is five.

To evaluate if the SNPs distribute equally throughout the pig chromosomes the exact locations of unique SNPs predicted on chromosome 1,4,7,14 were determined by alignment. A total of 1783 SNPs that mapped uniquely were plotted along these chromosomes as shown in Figure 2.

### Analysis of base changes

The SNPs in the subsets of candidate SNPs in which identical nucleotide substitution is represented in one, two, three or four sequences in the alignment were categorized according to nucleotide substitutions: C/T or G/A (transitions) and C/G, A/G, C/A, T/G (transversions). For each category, we calculated the relative nucleotide substitution frequencies for our SNP dataset and for the genomic porcine SNPs recorded in dbSNP [4] (Table 3). For the SNP subset in which identical nucleotide substitution is represented twice in the alignment, we observed a very similar relative increase in the proportion of transitions over transversions compared to the SNPs in dbSNP [4].

**Table 3:** Comparison of substitution frequencies of SNPs deposited in dbSNP [4] and polymorphisms identified in shotgun sequences.

			**Shotgun sequence analysis**			
	**dbSNP (genomic)**		**SNP redundancy = 1**		**SNP redundancy = 2**	
Transitions	5404	73,04%	64167	65,38%	4676	73,36%
Transversions	1995	26,96%	33984	34,62%	1698	26,64%

Total	7399		98151		6374	

### SNPs in common with dbSNP

To estimate whether SNPs predicted by our method are already present in the public database of dbSNP [4], we compared the two datasets by clustering. In dbSNP [4], we selected genomic SNPs (class = 1) with at least 50 bases of sequence on each side. These 7,896 SNPs were trimmed to have exactly 50 bases of flanking sequence and were analyzed for redundant records. The confirmed 7,586 unique SNPs were compared to our 98,151 predicted SNPs (singe representation of nucleotide substitution in alignment) by clustering. No clusters were formed, indicating that our dataset and dbSNP [4] share no SNPs in common.

### Experimental validation of candidate SNPs

To balance the sequence context and the number of times a polymorphism is represented in the sequence alignment, SNPs in which a nucleotide substitution was represented at least twice in the sequence alignment were chosen for experimental validation. A total of 163 selected candidate SNPs were validated by genotyping in a panel of three purebred boar lines (+ wild boar). A total of 61,777 genotype analyses were performed providing, in addition to SNP prediction validation, insights into allele frequencies that will be valuable information for association mapping and QTL studies. To measure the performance of our analyses, validated SNPs were included that previously had been used within the European Union (EU) pig biodiversity project II (Pig-BioDiv II) [24] as well as SNPs described by Rohrer et al [25] (Table 4). Also, 16 known SNPs in the IGF2-region [see Additional file 1] and 14 SNPs described in a number of publications [see Additional file 1] were included.

**Table 4:** The performance statistics for each source of SNPs tested in our animal panel.

**SNP source**	**Total**	**Fraction monomorph**
PigBioDiv	99	0,07
Rohrer et al.	39	0
Various Literature	14	0,07
IGF2-region	16	0
This study	163	0,18

For all 331 SNPs, the allelic variation was determined in our animal panel. In 29 cases, the predicted candidate SNPs turned out to be monomorphic. Smaller fractions of SNPs are observed to be monomorphic in the PigBiodiv and Various Literature SNP sets. The SNPs described by Rohrer et al [25] and the IGF2-region were all polymorphic in our animal panel.

For each predicted candidate SNP that appeared to be polymorphic in our panel, minor allele frequencies per boar line and overall average minor allele frequencies were calculated [see Additional file 2].

## Discussion

Because of their highly automated high-throughput assays, SNPs are the marker of choice for molecular genetic analysis. SNPs can be obtained cost effectively by analysing public sequence data sets [26-28]. When sequence trace files are involved at the identification of SNPs, true polymorphisms can be distinguished from sequencing errors. Polymorphisms in which the identified base is doubtful due to a high error probability in the trace file, and therefore the most probable cause of the observed variation, are filtered out [29-31]. The number of sequences in which a polymorphism is represented provides information as to whether a predicted SNP represents a true polymorphism. By filtering the observed sequence variation for polymorphisms in which the minor allele is represented at least twice in the sequence alignment, the chance that the predicted SNP is caused by sequencing errors is extremely small. Because the dataset used in our analysis consisted of shotgun sequences providing a 0.66× coverage, the sequence redundancy in our dataset is limited. This low genome coverage made it likely to detect true genetic variation already at a low sequence depth. Even SNPs with a single representation in the sequence alignment might represent true nucleotide polymorphism at this low genome coverage. However, the chance that SNPs with a single representation in the sequence alignment turns out to be monomorphic in a genotyping assay is relatively high. In order to obtain a set of high quality SNPs, we raised the threshold to a two times representation of a nucleotide substitution in the sequence alignment. A further increase of the representation constraint at this low genome coverage would lead to a SNP set in which the majority of genetic variation being detected is located in repetitive sequences. In these repetitive sequences, the degree of periodicity in nucleotide usage is high, making it hard to distinguish true allelic variation from predicted sequence variation caused by paralogous sequences. The over-representation of SNPs in repetitive sequences can be explained by errors in clustering paralogous repetitive sequences, as wel as by the 1.8 times higher SNP density in periodic DNA, which is observed in humans [32].

Although sequence quality scores and a redundancy-based approach were used to filter sequencing errors from true nucleotide polymorphisms, a non-random distribution of polymorphisms might occur in a particular dataset. These artefacts become visible when SNP statistics are compared to other SNP collections in the same species and are comparable to those found in related species. When compared to porcine SNPs deposited in dbSNP [4], our predicted SNPs in which a nucleotide substitution is represented at least twice in the sequence alignment show a similar transition/transversion ratio (Table 2). However, the transition frequency in humans was determined to be 60 to approximately 66% in vivo [16,6] and 60%–69% in silico [27,29], respectively. According to the SNP statistics in Table 1, it is evident that the transition/transversion ratio is highly biased by the fraction of SNPs in repetitive sequences in a particular dataset. A similar transition/transversion ratio for porcine SNPs deposited in dbSNP and our subset of SNPs, in which nucleotide substitutions are represented at least two times, is more likely explained by coincidence than being representative of the pig genome. The 0.6 fraction of sequences tagged as being repetitive in our SNP subset has likely influenced the transition/transversion ratio. Therefore the transition/transversion ratio observed in the total number of predicted SNPs, single redundancy, is likely more representative for the whole pig genome. This suggests a comparable transition/transversion ratio between humans and pigs, which was expected because of the evolutionary relatedness of these species.

A comparison of our collection of predicted candidate SNPs to the porcine SNPs in dbSNP [4] revealed no SNPs in common, not to our surprise. The average SNP density in the 2.7 Gb pig genome is estimated to be one in 336 base pairs [11], indicating that only a small fraction of the expected total of tens of millions of SNPs has been identified in the pig.

Not all predicted candidate SNPs turned out to be polymorphic in the animal panel. This doesn't implicitly mean that this 0.18 fraction (Table 4) includes falsely predicted polymorphisms. SNPs in the PigBioDiv [24] and the SNPs derived from various literature [see Additional file 1] that were previously experimentally validated resulted in (0.07) fractions of monomorphic SNPs. These fractions of monomorphic SNPs observed in this study can be explained by difference in selection of the animal panel on which the SNPs have been validated and the animal panel we used, as well as the absence of Chinese breed genetic background, near absence of Meishan and the use of another Large White in our panel.

Within our breed panel, we observed very low (<5%) Minor Allele Frequencies (MAF) in predicted candidate SNPs [see Additional file 2] and in the IGF2-region (data not shown). For SNPs in the IGF2-region, these low MAF are the result of intensive selection on that genomic region, whereas for the predicted candidate SNPs we did not know what to expect because of the unknown genomic location of these SNPs. Intensive selection also might have caused these very low MAF.

## Conclusion

The overall performance of the SNPs identified by our genome shotgun sequence mining approach is comparable to those available in existing SNP repositories. In perspective of the ongoing sequencing of the pig genome, the SNP data generated by this approach will provide a growing number of available markers that can be applied for genotyping and will increase the SNP marker density on the pig genome.

## Methods

### DNA sequence data

The entire genome shotgun sequences used in this study were downloaded from the NCBI Trace repository (species SUS SCROFA, center SDJVP). For all sequences, breed and mate pair information was obtained and stored in a relational database. Finished and unfinished BAC sequences obtained within the porcine genome sequencing project were retrieved from the SangerInstitute FTP site at .

BAC-end sequences were downloaded from the Ensembl [20] FTP site at .

BAC naming and mapping data were obtained from 

and , respectively. Naming and mapping data were stored in a local relational database.

### Clustering and alignment

Whole genome shotgun sequences were masked for mammalian-specific repeats and low complexity regions using RepeatMasker version open-3.1.7 [33] with options -xsmall, -species pig, default sensitivity and using the RepeatMasker Database release 20071204.

Clustering of data was performed by aligning the whole genome shotgun reads to the BAC sequences and BAC-end sequences using MegaBlast 2.2.16 [34].

Shotgun reads were aligned to BAC sequences using the alignment parameters -U T -s 122 -p95 -F m. Results were filtered for alignments with more than 90% of the shotgun sequence length. To reduce the amount of ambiguous results in the clustering, only alignment results with a bitscore >90% of the best scoring alignment for that shotgun sequence were stored in a relational database.

Clustering of shotgun sequences by alignment to BAC-end sequences was followed by assembling each cluster using CAP3 [35]. MegaBlast [34] parameters (-p 95 -s 32 -F m -U T) were matched to the CAP3 [35] settings (-o40, -p95), allowing only perfect assembled clusters. BAC-end sequences that were extended by shotgun sequences in the previous step were again used to cluster other shotgun reads until no extension occurred. Clustering results were stored in a local relational database.

Using the BAC and BAC-end naming and mapping information, we were able to query our clustering results by fingerprint contig name as used in the porcine physical map provided by the Sanger Institute. Per fingerprint contig the shotgun sequences that clustered to this region were selected and mate pairs were added using the mate pair information in the NCBI Trace repository.

Multiple sequence alignments of the selected shotgun sequences and their mate pairs were generated by the sequence assembly script PhredPhrap [21-23]. Shotgun sequence trace files were used as input for PhredPhrap [21-23], which was run using the default parameters.

### SNP identification

For identification of SNPs in the multiple sequence alignments of the shotgun sequences, we used PolyPhred [15] version 6.11 with options -snp hom -f 50, which lists homozygous SNPs with 50 bp flanking sequence.

Polyphred results were parsed into tables, information from which breed a sequence was derived and whether the SNP is located within a suspected repetitive sequence was added.

### Elimination of redundancy in identified SNPs by clustering

To remove any redundancy in our SNP predictions, the results (SNP position flanked by 50 bp genomic sequence) were first stored in FASTA format. The actual clustering was performed using blastclust [36] with parameters -S 99.5 -L 1.0 -b T -p F -F F. These parameters were also used to compare our SNP prediction results to public SNPs in dbSNP [4].

### Distribution of SNPs over pig chromosomes

Unique SNPs flanked by 50 bp genomic sequence predicted on chromosome 1,4,7,14 were mapped on the corresponding chromosomal sequence as provided by pre-ensemble [19]. The alignment was performed using BLAT [37] with the default parameters. SNPs that aligned uniquely to the chromosome with at least a 0.9 fraction of the flanking sequence involved in the alignment and a minimal sequence similarity of 96% were used to generate a SNP distribution plot.

### SNP validation

For SNP validation, 163 SNPs were selected, with regions covered by at least 4 reads and with a minimum SNP redundancy score of 2. These SNPs were subsequently genotyped in an animal panel consisting of three purebred boar lines that originated from (1) Duroc and Belgian Landrace, (2) Large White, (3) German Pietrain and (4) Wild Boar. The four lines included 129, 120, 109 and 21 individuals, respectively. Genotyping was performed using the Illumina GoldenGate(R) Genotyping assay on an Illumina^® ^BeadStation with veraCode(TM) technology. Oligonucleotides were designed, synthesized and assembled into oligo pooled assays (OPA) by Illumina Inc. Typing was carried out in a multiplex reaction, which included 384 loci.

## Availability and requirements

The SNPs identified in this study, in which the polymorphism was represented twice in the sequence alignment, have been deposited in the National Center of Biotechnology (NCBI) SNP database (dbSNP) under submitter handle WU_ABGC. NCBI_ss 106817370–106823609 represent predicted SNPs that were not tested on in our animal panel. Predicted SNPs that were confirmed are listed in [see Additional file 2]. SNPs with a single redundancy will be available on request.

## Authors' contributions

HHDK designed and developed the SNP prediction method and wrote the manuscript. AK and MdR designed and implemented the relational database. BD, SMK, RPC and SK collected and prepared the samples and performed the genotyping analysis. SK summarized the genotyping results. MAMG coordinated and supervised the experiment implementation, and assisted in the manuscript preparation. All authors read and approved the final manuscript.

## Supplementary Material

Additional File 1 **Supplementary information**. References to published SNPs used in this study. Click here for file

Additional File 2 **Validation of candidate SNPs by genotyping in a panel of three purebred boarlines and wildboar**. Listed are the 134 out of 163 predicted SNPs that show to be polymorphic in our animal panel. For all SNPs minor allele frequencies (MAF) were calculated in each purebred boarline and wildbore. From the obtained MAFs the average minor allele frequency was calculated. Click here for file
