# Supplementary material for: Mining for single nucleotide polymorphisms in pig genome sequence data
Source: BMC Genomics. 2009 Jan 6;10:4. doi: 10.1186/1471-2164-10-4 (PMC2637895; doi:10.1186/1471-2164-10-4)
Supplement: Additional File 1 — Supplementary information. References to published SNPs used in this study. [file 1471-2164-10-4-S1.doc]

# Supplementary information

**SNPs Various Literature**

1. Lin Z, Lou Y, Peacock J, Squires EJ: **A novel polymorphism in the 5' untranslated region of the porcine cytochrome b5 (CYB5) gene is associated with decreased fat androstenone level.** *Mamm Genome* 2005,**16**:367-373.

2. Buske B, Sternstein I, Reissmann M, Brockmann G: **Detection of novel single-nucleotide polymorphisms (SNPs) in the CYP21 gene and association analysis of two SNPs for CYP21 and ESR2 with litter size in a commercial sow population.** *J Anim Breed Genet* 2006,**123**:343-348.

3. Kollers S, Day A, Rocha D: **Characterization of the porcine FSCN3 gene: cDNA cloning, genomic structure, mapping and polymorphisms.** *Cytogenet Genome Res* 2006,**115**:189-192.

4. Grunwald KA, Schueler K, Uelmen PJ, Lipton BA, Kaiser M, Buhman K, Attie AD: **Identification of a novel Arg-->Cys mutation in the LDL receptor that contributes to spontaneous hypercholesterolemia in pigs.** *J Lipid Res* 1999,**40**:475-485.

5. Mackowski M, Szymoniak K, Szydlowski M, Kamyczek M, Eckert R, Rozycki M, Switonski M: **Missense mutations in exon 4 of the porcine LEPR gene encoding extracellular domain and their association with fatness traits.** *Anim Genet* 2005,**36**:135-137.

6. Kim KS, Larsen N, Short T, Plastow G, Rothschild MF: **A missense variant of the porcine melanocortin-4 receptor (MC4R) gene is associated with fatness, growth, and feed intake traits.** *Mamm Genome* 2000,**11**:131-135.

7. Wyszyńska-Koko J, Pierzchała M, Flisikowski K, Kamyczek M, Rózycki M, Kurył J: **Polymorphisms in coding and regulatory regions of the porcine MYF6 and MYOG genes and expression of the MYF6 gene in m. longissimus dorsi versus productive traits in pigs.** *J Appl Genet* 2006,**47**:131-138.

8. Mikawa S, Morozumi T, Shimanuki S, Hayashi T, Uenishi H, Domukai M, Okumura N, Awata T: **Fine mapping of a swine quantitative trait locus for number of vertebrae and analysis of an orphan nuclear receptor, germ cell nuclear factor (NR6A1).** *Genome Res* 2007,**17**:586-593.

9. Grindflek E, Moe M, Taubert H, Simianer H, Lien S, Moen T: **Genome-wide linkage analysis of inguinal hernia in pigs using affected sib pairs.** *BMC Genet* 2006,**7**:25.

10. Ciobanu D, Bastiaansen J, Malek M, Helm J, Woollard J, Plastow G, Rothschild M: **Evidence for new alleles in the protein kinase adenosine monophosphate-activated gamma(3)-subunit gene associated with low glycogen content in pig skeletal muscle and improved meat quality.** *Genetics* 2001,**159**:1151-1162.

11. Esteso G, Estellé J, Pérez-Enciso M: **Assignment of RAD51C to porcine chromosome 12 and identification of intronic variability**. *Anim Genet* 2005,**36**:461-462.

12. Nonneman D, Rohrer GA, Wise TH, Lunstra DD, Ford JJ: **A variant of porcine thyroxine-binding globulin has reduced affinity for thyroxine and is associated with testis size.** *Biol Reprod* 2005,**72**:214-220.

**SNPs IGF2**

* = SNPs were identified from the alignment of: AY242098-AY242112

ss<NUMBER> = SNPs from dbSNP

**SNPs PigBioDiv[24]**

20040204C-000053-3.0

20040204C-000058-2.0

20040204C-000058-3.0

20040204C-000238-3.0

20040204C-000275-1.0

20040204C-000370-3.0

20040204C-000375-2.1

20040204C-000382-3.0

20040204C-000466-2.0

20040204C-000546-1.0

20040204C-000559-1.0

20040204C-000900-3.0

20040204C-000934-1.0

20040204C-001008-1.3

20040204C-003766-5.0

20040204C-003781-3.0

20040204C-003789-8.0

20040204C-003855-6.0

20040204C-003935-1.2

20040204C-004106-1.2

20040204C-004422-3.0

20040204C-004650-2.0

20040204C-004766-1.0

20040204C-004766-1.2

20040204C-004766-1.3

BV079380.1CD59STS21.0

BV079389.1FSHBSTS21.0

BV079399.1P005E11STS11.0

BV079403.1P008B07STS11.18

TC161981-2.0

TC162058-6.0

TC162684-6.0

TC162752-9.0

TC162931-6.0

TC162975-1.2

TC162975-10.0

TC162975-9.0

TC163031-7.0

TC163039-13.0

TC163159-1.1

TC163355-3.0

TC163448-12.0

TC163452-10.0

TC163456-10.1

TC163582-15.0

TC163833-12.1

TC163867-7.1

TC164077-15.2

TC164255-7.0

TC164294-7.0

TC164365-5.0

TC164637-11.1

TC164655-2.3

TC164782-1.0

TC165224-1.3

TC165262-4.1

TC165338-2.1

TC165343-2.0

TC165511-15.0

TC165511-15.2

TC165777-9.2

TC166157-4.0

TC167503-6.0

TC181289-9.0

TC181500-3.0

TC181610-11.3

TC181642-1.0

TC181797-13.0

TC181810-4.0

TC181914-1.0

TC181955-4.0

TC181988-2.0

TC182022-4.0

TC182119-5.0

TC182134-30.0

TC182172-5.0

TC182189-5.0

TC182237-19.0

TC182410-4.0

TC182480-2.1

TC182480-3.0

TC182480-3.1

TC182491-8.0

TC182491-8.1

TC182609-10.2

TC182614-10.1

TC182614-11.0

TC182788-11.0

TC182889-3.0

TC183155-13.0

TC183250-1.0

TC183327-2.0

TC183423-4.0

TC183664-28.0

TC183697-2.0

TC184062-6.1

TC184157-2.1

TC185034-3.0

TC185453-6.0

**SNPs Rohrer et al. [25]**

MARC-0002

MARC-0003

MARC-0004

MARC-0006

MARC-0008

MARC-0009

MARC-0010

MARC-0011

MARC-0012

MARC-0014

MARC-0015

MARC-0017

MARC-0021

MARC-0022

MARC-0023

MARC-0024

MARC-0025

MARC-0026

MARC-0027

MARC-0028

MARC-0029

MARC-0030

MARC-0031

MARC-0032

MARC-0033

MARC-0034

MARC-0036

MARC-0037

MARC-0038

MARC-0040

MARC-0041

MARC-0042

MARC-0044

MARC-0047

MARC-0049

MARC-0050

MARC-0052

MARC-0054

MARC-0059
